# Computer-Aided Clinical Trial Recruitment Based on Domain-Specific Language Translation: A Case Study of Retinopathy of Prematurity

**DOI:** 10.1155/2017/7862672

**Published:** 2017-04-05

**Authors:** Yinsheng Zhang, Guoming Zhang, Qian Shang

**Affiliations:** ^1^School of Management and E-Business, Zhejiang Gongshang University, Hangzhou, Zhejiang 310018, China; ^2^Pediatric Retinal Surgery Department, Shenzhen Eye Hospital, Shenzhen Key Ophthalmic Laboratory, The Second Affiliated Hospital of Jinan University, Shenzhen, Guangzhou 518040, China; ^3^Management School, Hangzhou Dianzi University, Hangzhou, Zhejiang 310018, China

## Abstract

Reusing the data from healthcare information systems can effectively facilitate clinical trials (CTs). How to select candidate patients eligible for CT recruitment criteria is a central task. Related work either depends on DBA (database administrator) to convert the recruitment criteria to native SQL queries or involves the data mapping between a standard ontology/information model and individual data source schema. This paper proposes an alternative computer-aided CT recruitment paradigm, based on syntax translation between different DSLs (domain-specific languages). In this paradigm, the CT recruitment criteria are first formally represented as production rules. The referenced rule variables are all from the underlying database schema. Then the production rule is translated to an intermediate query-oriented DSL (e.g., LINQ). Finally, the intermediate DSL is directly mapped to native database queries (e.g., SQL) automated by ORM (object-relational mapping).

## 1. Introduction

Clinical trials (CTs) are the building blocks for evidence-based medicine (EBM). Clinical trials are typically performed under the guidance of clinical trial protocols (CTPs), which are standardized guidelines for conducting CT. A CTP contains several components, including purpose, study design, recruitment criteria of subjects, treatment of subjects, assessment of efficacy, assessment of safety, adverse events, quality control and assurance, and ethics. Among them, “recruitment criteria” (or “eligibility criteria”) is an essential step for conducting CTs.

The recruitment criteria specify a set of common features that define the population subset of interest. These features include age, gender, habit, diagnosis, stage of disease development, surgery history, and genetic data. The recruitment criteria usually contain both “inclusion” and “exclusion” rules, which define required and unwanted features, respectively. For certain CTs, the criteria could be quite complex, making the patient enrollment very challenging. With the rapid development of healthcare IT (HIT), electronic patient data acquired during the clinical care process offers a new approach to facilitate the recruitment procedure.

Much work has been reported concerning the previously mentioned approach. The traditional way of this approach usually relies on DBA (database administrator) or clinical engineers. The criteria are initially written in natural language form by CT protocol authors or clinical researchers. Then, the engineers translate the narrative criteria into native database query language, such as SQL. Due to the professional barrier and possible ambiguity of natural language, such criteria translation can be inaccurate. Secondly, the involvement of engineers has increased both human resource and communication cost. For these reasons, some researchers chose to develop computer-aided tools or clinical trial recruitment support systems (CTRSS) to facilitate the recruitment procedure. The following manuscript will introduce some of the related work.

## 2. Related Work

European researchers built the TRANSFoRm Query Workbench Tool [1] to author, store, and execute clinical data queries to identify potential subjects for clinical studies. This tool can create recruitment criteria in computable representations. Then the criteria are translated into executable queries in institution-specific databases. The Query Workbench Tool uses the Clinical Data Integration Model (CDIM) [2] as an intermediate standard ontology, so it can support queries across multiple heterogeneous data sources. The UK CancerGrid [3] project designs a widely accepted clinical trial model by controlled vocabulary and common data elements (CDEs). Based on this model, a cancer data query system has been developed which supports data sharing across CancerGrid-compliant clinical trial boundaries. Penberthy et al. [4] designed a CT matching system. The system allows users to define the recruitment criteria through a form-based GUI tool. Then the system will perform periodical automatic screening against patients in the information system. Matched subjects will be sent to researchers via emails. The BreastCancerTrials.org [5] project creates a CT matching website whose targeting audience is patients. It uses self-reported patient data collected by web-based forms to match against existing registered cancer CTs and recommend patients with available CTs. Patients can also redirect themselves to participating research sites. BreastCancerTrials.org can increase patients' awareness to participate cancer CTs, and can improve patient data usage among multiple CT research groups. There are also several experimental researches that use more sophisticated methods, such as natural language processing (NLP) [6] and semantic web [7], to facilitate the automatic extraction and comparison of recruitment criteria and patient data.

Generally speaking, much research work focuses on querying across heterogeneous sources, and uses form-alike structured data entry (SDE) techniques to author recruitment criteria. These SDE tools are usually designed according to some intermediate information model (or ontology). Based on this model, the recruitment criteria and referenced data elements can be defined in formal representations, which have the unique advantage in data exchange and semantic interoperability. However, this paradigm requires mapping between the intermediate model and individual databases when performing patient queries. Such mapping work can be quite knowledge-intensive and time-consuming, and due to information granularity and semantic differences, the mapping of certain data elements can be extremely difficult or even unsupported.

With such concern, this paper tries an alternative method that uses direct syntax translation to avoid model mapping. The following manuscript will introduce the method and a corresponding case study in detail.

## 3. Method

### 3.1. Knowledge Representation of Clinical Trial Recruitment Criteria

The first step of this study is the knowledge representation of recruitment criteria and relevant patient data. An analysis on knowledge representation will help to determine whether a certain kind of information model or formal language has the ability to represent all related entities (e.g., concepts and rules) in this domain. Following are two typical recruitment criteria used in clinical trials. These two examples are related to retinopathy of prematurity (ROP), which is the target disease in our case study.

***Example 1.* Consider the following:**

**Table:** 

[Aim of CT]
Assess the anti-neovascularization activity of intravitreal bevacizumab, as determined by regression of neovascular vessels of retinopathy of prematurity (ROP), in neonates with acute stage 3 ROP in zone I or posterior zone II with plus disease.
[Recruitment criteria]
Birth weight ≤ 1500 grams
Gestational age ≤ 30 weeks
Diagnosis = stage 3 ROP in zone I or posterior zone II
Drug use = bevacizumab (Avastin®)
*Without* congenital systemic anomaly
*Without* congenital ocular abnormality.

***Example 2.* Consider the following:**

**Table:** 

[Aim of CT]	
Assess Pan-VEGF (vascular endothelial growth factor) blockade for the treatment of retinopathy of prematurity.	
[Recruitment criteria]	
Gestational age ≤ 30 weeks	
Gestational age ≤ 36 1/7 weeks	
Diagnosis = type 1 pre-threshold ROP	
No prior treatment	
*Without* media opacity precluding fundus visualization	
*Without* ocular or periocular infection(s).

As seen from the examples, typical CT recruitment criteria consist of a set of medical concepts (e.g., birth weight, gestational age, and diagnosis) and a rule condition composed of these concepts (e.g., Birth weight ≤ 1500 grams && Gestational age ≤ 30 weeks && (Diagnosis = stage 3 ROP in zone I || Diagnosis = posterior zone II) ). In this study, we chose the production rule as the knowledge representation for CT recruitment criteria. In runtime, the data sources should feed the rule with actual patient data for succeeding query or reasoning.

### 3.2. Patient Query for CT Recruitment by Syntax Translation

Clinical data can be conveyed in two styles. One is natural language (NL), which is extensively used by humans in daily life. The other is formal language (FL) or domain-specific language (DSL), which is usually designed for a specific purpose and contains a limited set of symbols and syntax. Typical FLs/DSLs include the Arabic numbers and arithmetic symbols used in mathematics, the molecular formula in chemistry, and programming languages. The data carried by NL are usually “unstructured” while the data conveyed by FL/DSL are mostly “structured.” Though FL/DSL may lack expressiveness than NL, they are much more suitable for computers to parse and process.

The essence of patient query in CT recruitment is the translation of NL (recruitment criteria from CTP or clinical researchers) to FL/DSL (e.g., SQL). Although there is related work that uses NLP techniques to facilitate the translation of NL to FL/DSL, there is a gap between research and real application. NLP (especially for Chinese NL) is still premature and many studies use the more reliable SDE instead. In these studies, the recruitment criteria are first authored in certain formal representation by SDE. Next, the formally represented criteria need to be converted to database-specific queries (e.g., SQL). As mentioned before, many studies depend on a sharable model or ontology and have to handle the mapping between the sharable model and individual data sources. Such a mapping work can be a quite complicated, knowledge-intensive, and time-consuming task. As an alternative, this paper proposed a method based on DSL syntax translation, other than ontology mapping.

Our method directly uses the underlying database's schema as a reference model for the SDE tool and its generated production rules. That is, all rule variables in the SDE tool come from data fields in the database schema. For example, the SDE tool outputs a production rule “[BirthWeight] < 1.0 && [GestationalAgeInWeeks] < 30”. In this rule, the “[BirthWeight]” and “[GestationalAgeInWeeks]” rule variables have corresponding “BirthWeight” and “GestationalAgeInWeeks” data fields in the underlying database schema. The next step is to convert such production rules to query-oriented DSL. The query-oriented DSL is provided in many modern programming languages, such as Java, C#, and VB.Net. Take LINQ (language-integrated query) as an example. In Microsoft .Net Framework, LINQ is a language subset of C# and VB.Net. LINQ supports nearly 40 operators, including “select,” “from,” “in,” “where,” and “order by.” With LINQ, programmers can directly write SQL-style C# or VB.Net codes to query underlying data providers, such as ORM (object-relational mapping), ODBC (open database connect), or XML (extensible markup language) files. Because our case study is conducted in the context of Microsoft.Net framework and the SQL server relational database, we will use LINQ for demonstration purposes. For other technical platforms, there are also equivalent technologies, such as JINQ, Linq4j, and JaQue, for the Java platform. After the production rule is converted to LINQ, LINQ to native-SQL conversion is automatically supported by the ORM provider, and no extra effort is required.

In the previously mentioned process, the production rule, LINQ, and SQL are all strictly constrained FLs/DSLs, so the syntactic conversion between them is explicit and unambiguous. In addition, these DSLs all use the underlying database schema as the reference model, so there is no need for a complex concept mapping. Such concept mapping is a well-known issue in medical informatics, aka the “curly braces” problem [8], which involves mapping external clinical data to rule variables in the rule expression.

**Figure 1:**
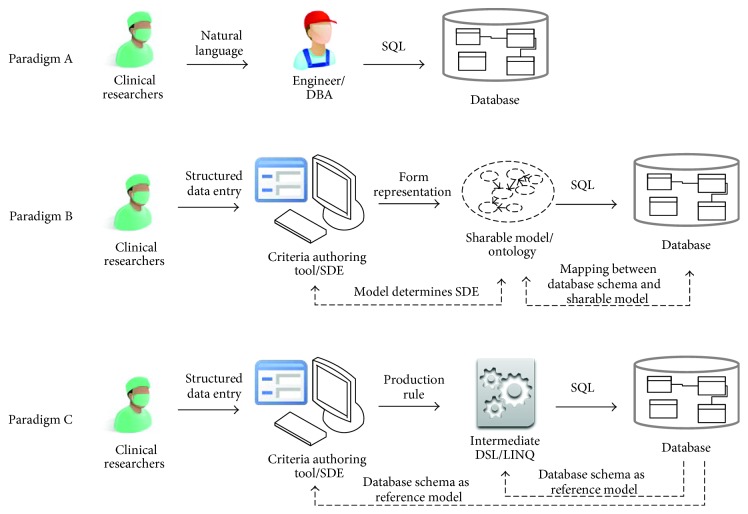
Comparison of the patient query paradigms used in clinical trial recruitment (demonstrated in the case of relational database). Paradigm A depends on clinical engineers or DBA to translate clinical researchers' natural language representation of recruitment criteria into database-specific query language, for example, SQL. Paradigm B uses SDE tools to author formally represented criteria based on a sharable model. The criteria are then translated to SQL by the underlying mapping between the sharable model and individual database. Paradigm C uses SDE to author recruitment criteria in the form of the production rule, which is then translated into LINQ syntax. LINQ to SQL conversion is naturally supported by ORM-like technologies. DBA = database administrator; SDE = structured data entry; DSL = domain-specific language; LINQ = language-integrated query; SQL = structured query language; ORM = object-relational mapping.

Figure 1 illustrates the comparison of different paradigms. Paradigm A depends on DBA to translate clinicians' narrative recruitment criteria to native database queries. As mentioned before, such manual translation can sometimes be inaccurate due to professional barrier and the ambiguous nature of NL. Paradigm B uses SDE to author formally represented criteria based on a sharable model. The formally represented criteria are then translated to SQL by underlying mappings between the sharable model and individual database. Paradigm C, which is proposed in this paper, also uses SDE to author recruitment criteria in the form of the production rule, but does not involve any data mapping jobs. In the following manuscript, we will introduce a case study to demonstrate our method and give more details on system implementation.

## 4. Case Study and System Implementation

### 4.1. Clinical Settings

The case study is conducted in Shenzhen Eye Hospital, which is a 200-bed class III specialized hospital in China. Since 2013, we have been developing a ROP (retinopathy of prematurity) management system for the pediatric retinal surgery department. ROP, aka Terry syndrome, is a common eye disease for prematurely born babies, especially those with low birth weight and early gestation age. ROP is related with disorganized growth of retinal blood vessels resulting in retinal scarring or detachment. Without early screening and timely intervention, ROP can lead to severe visual impairments or even blindness. ROP has become a major reason for children blindness. It is estimated that China has at least 0.2 million ROP newborns each year. ROP has now become a nonneglectable problem in China, whereas ROP-oriented information systems are scarce on the Chinese market. Under this circumstance, we built this ROP management system to help clinicians to manage ROP screening information and track patient disease development.

The ROP management system is also designed as a regional telemedicine system. It covers not only the ROP screening cases in Shenzhen Eye Hospital, but also those from partner hospitals, such as Shenzhen Dapeng-District Maternity Hospital, Meizhou People's Hospital (Guangdong Province, China), and Puning People's Hospital (Fujian Province, China). These partner hospitals can upload ROP-related data (e.g., RetCam imaging, newborn info) to central data repository via the system. Then the ROP medical team in Shenzhen Eye Hospital will help to diagnose these cases. Until now, the system has enrolled more than 22,238 patient cases. The demo version of the system is http://ropd.brahma.pub.

With the advent of “big data era,” how to effectively use the collected data has become a great concern for both clinicians and researchers. Supporting clinical trial (CT) is one of the typical applications of clinical data.

### 4.2. Computer-Aided CT Recruitment

**Figure 2:**
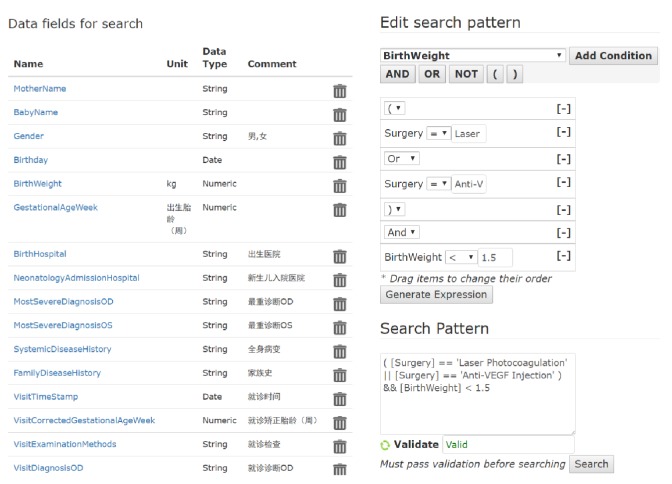
SDE for authoring CT recruitment criteria. The left panel is a list of data fields that can be used as rule variables in the search patterns. These data fields come from the database schema of the central data repository. The right panel is a graphical rule editor. It supports conditional predicates (composed of rule variables and comparison operators, e.g., [BirthWeight] <= 1.5), logical operators (AND, OR, and NOT), and parentheses (specify precedence). For each rule variable, the available comparison operators are strictly confined based on its data type. If the variable is numeric or date type, allowed comparison operators will be “>=, <=, >, <, =, !=”. If the variable is string type, the allowed comparison operator will be “=,” and the rhs (right-hand side) constant can be a string literal with wild cards (“%”). The sequence of rule components can be adjusted by drag and drop. Furthermore, the graphical editor can generate a rule expression into the textual editor. The textual editor also allows the user to directly edit rule expression with autocomplete (IntelliSense) utilities.

Based on Paradigm C proposed in Figure 1, we developed a computer-aided CT recruitment module as a subsystem in the ROP management system. The kernel idea of this system is to reuse the patient data in the central data repository for clinical research purposes. As mentioned before, the CT recruitment criteria vary from study to study. To fulfill different requirements, we developed a versatile SDE tool (Figure 2) by which clinicians can author customized search patterns. The output of the SDE tool are production rules, as the formal representation of CT recruitment criteria.

**Figure 3:**
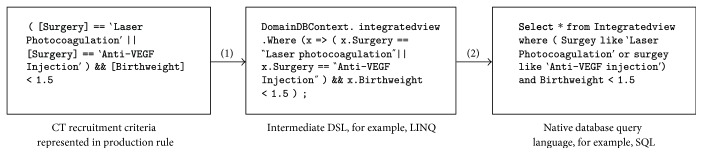
Example of converting production rule to native database query. (1) First, the production rule is translated to an intermediate DSL, such as LINQ. (2) Then, the LINQ DSL is translated to native database query automated by the ORM module.

After the production rule is generated, it needs to be converted to native queries towards the underlying data repository.  Figure 3 gives a concrete example of this conversion process. In Figure 3, the production rule is first translated to LINQ lambda expressions (the anonymous function inside the “Where” clause). Then, the LINQ is automatically translated to a corresponding native SQL query by ORM. It should be noted that both the LINQ and SQL code snippets in Figure 3 use only one entity set/data table: “IntegratedView.” In reality, patient-related data are often dispersed among multiple data tables. For example, in our ROP management system, there are “patient master table,” “patient visit table,” and “patient surgery table.” Sometimes, users have to make joint queries between multiple tables. In order to reduce complexity, we created a database view “IntegratedView” by joining existing tables. Although “IntegratedView” is a “virtual table,” it is a fully functioning searchable object just as physical tables are, and it is equally supported by ORM. Such a view not only integrates data from multiple tables, but also reduces complexity by providing a unified logical schema. This schema is referenced by all modules across the systems, including the following: (1) the data fields (Figure 2) used to compose production rules; (2) the entity set (“domainDBContext.IntegratedView”) and entity properties (e.g., “x.Surgery,” “x.BirthWeight”) in LINQ; and (3) and the final SQL code transformed by ORM.

Based on the DSL-conversion process, the original CT recruitment criteria represented in production rule has been ultimately converted to a native SQL statement on the underlying database. The returned patients will be shown as an html table. Users can export the search result into Excel or csv (comma-separated value) files. They can also check each individual patient, and assign a specific “CT research tag” to this patient.

### 4.3. Evaluation

The CT recruitment system was first brought on line in August 2015. Until now (November 2016), system logs show there have been 230 user queries. Although we have not yet conducted a large-scale scientific evaluation on the system, we have collected plenty of feedbacks from both end users and clinical engineers. Based on these feedbacks, several aspects of the system can be evaluated.

*(1) Implementation Cost.* As mentioned before, the criteria authoring SDE tools used in many related works are based on a shared information model or ontology, and the mapping of the shared model to native database queries can be quite complicated and time consuming. The method proposed in this paper does not use such an intermediate model, so the mapping effort is saved and the implementation cost is relatively low. In addition, this method uses LINQ as an intermediate DSL. Because both LINQ and the recruitment criteria production rules are strictly constrained DSLs, the conversion between them is unambiguous and easy to achieve. What is more, LINQ can use ORM as the data provider, so the conversion between LINQ and SQL can be automated by ORM.

*(2) DSL Expression Power.* In this study, the SDE tool uses production rule to author CT recruitment criteria. The expression power of the DSL determines how well the criteria can be represented and processed by the information system.  Table 1 shows the operators and data types used in the production rule DSL. Although the DSL only supports a limited set of operators (e.g., it does not support assignment “=” and arithmetic operators “+, -, ∗, /”), it has satisfied the CT recruitment requirements in 230 query tasks since its first deployment. The expression power of this DSL lies in two aspects: (1) Complex rules can be composed by the combination of fundamental primitives. (2) The wild card supported in this system effectively handles the free-text data fields. In the production rule DSL, “%” represents any text and can be used to compose complicated literal patterns. This wild card is natively supported by the underlying SQL engine.

*(3) Performance.* A quantitative evaluation of the system performance is carried out on a cloud-computing virtual machine (CPU: Dual Core 2.39 GHz, RAM: 4 GB, OS: Windows Server 2012 R2 Datacenter 64-bit Edition, DB: SQL Server 2012 Express). The total consumed time of a CT recruitment query task contains 4 parts: (1) Rule validation. The system first checks whether the rule expression can be parsed as a well-structured tree (using the ANTLR library). (2) DSL translation: “production rule –> LINQ –> SQL.” (3) SQL query: the query execution time by the underlying DB Server. (4) Client processing: time consumed by the web browser, including AJAX calls to the server side and html rendering (show server-returned records in web page).

Three CT recruitment rules in different complexity are tested. As seen from Table 2, the most time-consuming parts are the SQL query and client processing, while the rule validation and DSL translation cost little. It can also been seen that as the rule complexity grows up, the consumed time of the DSL translation process and the underlying SQL server does not scale up dramatically. This shows that the computer-aided CT recruitment system has a good performance curve in regard to rule complexity.

*(4) Rule Variable Extensibility.* After the first release of the CT recruitment system, one frequent request posed by end users is supporting more data fields (rule variables). In the beginning, we only provided a few basic data fields in the rule authoring tool, such as birthday, gestation age, birth weight, and diagnosis. However, as users conduct more CTs, they continually request to extend more data fields, such as family disease history, drug use during pregnancy, and CPAP (continuous positive airway pressure) treatment.

To meet these frequent requests, we developed the rule authoring SDE tool based on pure web-client technologies (HTML and JavaScript), and the rule authoring tool is decoupled from server-side rule parsing and execution logics. When needing to extend a new rule variable, clinical engineers only need to extend a few lines of HTML and JavaScript codes for the authoring tool. Because the rule variable name and data type are the same as the corresponding data field in the database, the recruitment criteria rule produced by the authoring tool can be directly parsed and executed by the successive modules without any further modification. In this sense, the rule variable extensibility is quite satisfying and the system maintenance workload is greatly reduced.

*(5) Query Accuracy and Recall Rate.* Accuracy and recall rate are the two common measures for assessing query systems. For our system, we found that these two measures depend on how well the users write the rule expressions. The system is just an “executor” of the search rule. As there is no NLP or fuzzy-logic module in the system that could bring “uncertainty” or “ambiguity,” the system itself does not compromise the overall query accurate and recall rate. By the rule authoring tool, the users can constantly improve query accuracy and recall rate by routine techniques, such as joining multiple variable conditions, increasing or decreasing thresholds, and adding more logical branches.

## 5. Conclusions and Discussions

The CT recruitment system has been used in Shenzhen Eye Hospital for more than a year. Clinical evaluation and feedback has proven its usability. The system is also well received by system developers and clinical engineers: the development and maintenance cost is significantly low, and the extensibility is also satisfying.

Such positive reviews are mainly due to two features of the system. (1) The system directly uses the underlying database schema as a reference model for all related modules, such as the rule authoring tool, the intermediate LINQ code, and the final SQL query. In this way, the system complexity and data-mapping efforts are greatly reduced. (2) The system uses the query-oriented DSL (e.g., LINQ in C# and VB.Net; JINQ, Linq4j, and JaQue in Java) as an intermediate formal language. Such DSL uses similar syntax as production rules and SQL, and are supported by ORM data providers. In this way, the translation process from recruitment criteria to native database queries is greatly simplified.

Compared to other related work, this study has several limitations and arguments.

*(1) Data Integration and Knowledge Sharing.* Much existing related work focus on how to achieve data sharing and data query across heterogeneous clinical data sources. The core method of these works is a common ontology or data model. For each data source, a middleware or data mapping module is provided to map the individual database to the shared model. The rule authoring tool also depends on the shared model, so the recruitment criteria are represented in a sharable computable form, and can be distributed as sharable knowledge assets.

On the other hand, because the ROP management system in our case study already has a central data repository that collects and stores data from multiple hospitals (in other words, the data integration work has been shifted down to the underlying data repository), we do not focus on the data integration issue from heterogeneous data sources. Our method does not need a shared ontology or data mapping middleware. However, the disadvantage is also apparent: the recruitment criteria rules created by the authoring tool are tightly coupled with the local database schema. In other words, the rules are “institution-specific” and are difficult to be distributed as sharable knowledge assets.

*(2) Extending Rule Variables.* As mentioned before, the rule variables provided by the rule authoring SDE tool come from the underlying database schema. In this way, the rule variables referenced in the rule expression can be “recognized” and “processed” by all related modules in the system. Such a design incurred some argument: What if the user wants to add a medical concept/rule variable that does not exist in current database schema? We can analyze this argument in 3 cases: (a) The concept is a high-level or coarse-grained concept, compared to the data fields in the database. For this case, the high-level concept can usually be composed by existing fine-grained items. For example, a user wants to use a “most severe diagnosis” concept, and the database has “most severe diagnosis OD” and “most severe diagnosis OS” data fields (OD = right eye, OS = left eye). A criteria rule of “[most severe diagnosis] = ROP_Stage4A” concept can be represented as “[most severe diagnosis OD] = ROP_Stage4A || [most severe diagnosis OS] = ROP_Stage4A”. (b) The concept is a low-level or fine-grained concept. It is usually more difficult and complex to handle this case. For example, the user wants to use “ROP zone” and “ROP stage” as rule variables, but the database only has a string-typed “diagnosis” data field. A rule of “[ROP zone] = 1 && [ROP stage] = 3” can be represented as “[diagnosis] = %Zone1Stage3% || [diagnosis] = %Stage 3 in zone 1%” (% is a wild card). However, due to the unconstraint nature of natural language, not all eligible patients can be covered by the previously mentioned rule, which will compromise the recall rate. Moreover, for many cases, it can even be impossible to compose an equal representation with coarse-grained data fields. (c) The concept is totally new to the current database. For example, the user wants to do some genotype research, but there are no genetic data fields in the underlying database. For these cases, the only solution in the context of our proposed method is to extend the database schema. In summary, case (a) is well supported in the system, case (b) is partially supported, and case (c) is not supported. For unsupported cases, the only feasible solution is to extend the database schema to add new data fields.

*(3) Make Use of Unstructured Clinical Data.* In this case study, the data stored in the ROP management system are mostly well formatted and structured. However, some relevant patient information still remains in the non-structured form (e.g., scanned handwritten RetCam report, and eye surgery record). How to use these narrative data is a long-existing problem faced by clinical researchers, and the promising NLP technologies have always been a hot research area. Joint query on both structured and nonstructured data will be a meaningful research topic for our CT recruitment system. Another kind of important nonstructured data is medical imaging. For ROP, the RetCam image is a very important evidence for diagnosis. How to extract morphological or even physiological information from the RetCam image is a challenging and meaningful task. Combining the imaging data will further improve the patient query result.

## Figures and Tables

**Table 1:** Language primitives of the production rule DSL.

Primitives	Type	Description
&&	Logical operator	Logical AND
||	Logical operator	Logical OR
!	Logical operator	Logical NOT
>	Comparison operator	Greater than
>=	Comparison operator	Greater than or equal
<	Comparison operator	Less than
<=	Comparison operator	Less than or equal
==	Comparison operator	Equal
!=	Comparison operator	Not equal
(	Operator precedence	Left parenthesis
)	Operator precedence	Right parenthesis
[	Variable identifier	Text between “[” and “]” is interpreted as a variable name
]	Variable identifier	
Boolean	Data type	For example, true/false
Numeric	Data type	For example, 1.0
Date	Data type	For example, “2016-12-25”
String	Data type	For example, “Stage 3 ROP in zone I”
%	Wild card	Wild card for string comparison

**Table 2:** Query performance result of the computer-aided CT recruitment system.

CT Recruitment rules in different complexity	Rule description	Rule validation	DSL translation	SQL query	Client processing	Total time
[BirthWeight] <= 1.0 && [GestationalAgeWeek] < 29	Find patients whose birth weight <= 1 kg and gestational age < 29 weeks	<1 ms	<1 ms	151 ms	285 ms	436 ms
[BirthWeight] <= 1.5 && [GestationalAgeWeek] <= 30 && [Birthday] >= 2014-01-01 && ([MostSevereDiagnosisOD] == %retin% || [MostSevereDiagnosisOS] == %retin% )	Find patients born after 2014 and whose birth weight <= 1.5 kg and gestational age <= 30 weeks and who have retina-related diseases	<1 ms	<1 ms	157 ms	289 ms	446 ms
[BirthWeight] <= 1.5 && [GestationalAgeWeek]<= 30 && [Birthday] >= 2014-01-01 && ( [NumberOfFetus] >= 2 || [Homozygotic] == true || [MotherAge] >= 35 || [OxygenMethod] == %oxygen% || [SystemicDiseaseHistory] == %Apnea% || ( [PregnancyStatus] == %HTN% || [PregnancyStatus] == %DM% || [PregnancyStatus] == %PIH%) || [PregnancyMedication] == %Corticosteroids% || ( [MostSevereDiagnosisOD] == %ROP% || [MostSevereDiagnosisOS] == %ROP% || [MostSevereDiagnosisOD] == %retin% || [MostSevereDiagnosisOS] == %retin% ) || ( [FamilyDiseaseHistory] == %retin% || [FamilyDiseaseHistory] == %fundus%) )	Find patients born after 2014 and whose birth weight <= 1.5 kg and gestational age <= 30 weeks and who meet one of the following conditions: homozygotic multiple birth, mother age >=35, oxygen therapy history, OSAS, having at least one of HTN, DM, or PIH during pregnancy, using corticosteroids during pregnancy, having retina-related diseases, or having family disease history of retina or fundus-related diseases	1 ms	<1 ms	159 ms	283 ms	443 ms

The results are averaged on 10 tests. Detailed result can be downloaded from http://ropd.brahma.pub/pages/20170130.xls.
